# Identifying inpatient hospitalizations with continuous electroencephalogram monitoring from administrative data

**DOI:** 10.21203/rs.3.rs-2882806/v1

**Published:** 2023-05-08

**Authors:** Marta Fernandes, M. Brandon Westover, Sahar F. Zafar

**Affiliations:** Massachusetts General Hospital (MGH); Beth Israel Deaconess Medical Center; Massachusetts General Hospital (MGH)

**Keywords:** Health services research, EMU, EEG, electronic health records, machine learning

## Abstract

**Background:**

Continuous electroencephalography (cEEG) is increasingly utilized in hospitalized patients to detect and treat seizures. Epidemiologic and observational studies using administrative datasets can provide insights into the comparative and cost effectiveness of cEEG utilization. Defining patient cohorts that underwent acute inpatient cEEG from administrative datasets is limited by the lack of validated codes differentiating elective epilepsy monitoring unit (EMU) admissions from acute inpatient hospitalization with cEEG utilization. Our aim was to develop hospital administrative data-based models to identify acute inpatient admissions with cEEG monitoring and distinguish them from EMU admissions.

**Methods:**

This was a single center retrospective cohort study of adult (≥ 18 years old) inpatient admissions with a cEEG procedure (EMU or acute inpatient) between January 2016-April 2022. The gold standard for acute inpatient cEEG vs. EMU was obtained from the local EEG recording platform. An extreme gradient boosting model was trained to classify admissions as acute inpatient cEEG vs. EMU using administrative data including demographics, diagnostic and procedure codes, and medications.

**Results:**

There were 9,523 patients in our cohort with 10,783 hospital admissions (8.5% EMU, 91.5% acute inpatient cEEG); with average age of 59 (SD 18.2) years; 46.2% were female. The model achieved an area under the receiver operating curve of 0.92 (95% CI [0.91–0.94]) and area under the precision-recall curve of 0.99 [0.98–0.99] for classification of acute inpatient cEEG.

**Conclusions:**

Our model has the potential to identify cEEG monitoring admissions in larger cohorts and can serve as a tool to enable large-scale, administrative data-based studies of EEG utilization.

## Background

Continuous electroencephalography (cEEG) is increasingly utilized in hospitalized patients with acute brain injury or altered mental status to detect seizures and other seizure-like patterns that can worsen outcomes (Herman et al. 2015). In the United States, there has been a 10-fold increase in the use of cEEG in acute inpatient setting, particularly in critical care (Hill et al. 2019; Ney et al. 2013). Detection of seizures and other seizure-like patterns on cEEG frequently results in anti-seizure medication (ASM) treatment escalation (Dhakar et al. 2022; Kilbride, Costello, and Chiappa 2009; Rossetti et al. 2020; Tabaeizadeh et al. 2020; Zafar et al. 2018). However, there is limited data on whether cEEG-guided ASM escalation improves outcomes (Sivaraju and Gilmore 2016). At the same time, cEEG is resource intensive with limited availability in smaller health care facilities, being utilized more frequently in larger, urban and academic centers (Hill et al. 2019; Ney et al. 2013). Epidemiologic studies and observational studies using large administrative datasets can provide insights into the comparative effectiveness and cost effectiveness of cEEG utilization in acutely ill patients, and guide policies and protocols that can improve access to cEEG for patients where indicated (e.g., identifying patients that may benefit most from transfer to centers performing cEEG), develop cEEG utilization quality measures, generate evidence for rigorous randomized trials on cEEG guided anti-seizure treatment, and ultimately improve outcomes.

Prior work examining administrative datasets has shown that cEEG monitoring in hospitalized critically ill patients is associated with lower in-hospital mortality (Hill et al. 2019; Ney et al. 2013). However, a limitation of prior studies that have used administrative datasets is the lack of validated codes differentiating elective epilepsy monitoring unit (EMU) admissions from acute inpatient hospitalization with cEEG utilization. Acute inpatient cEEG and EMU EEG have the same International Classification of Diseases (ICD) and Current Procedural Terminology (CPT) codes. As a result, prior work has excluded all patients that were elective admissions or were not mechanically ventilated to define patient cohorts that underwent acute inpatient continuous EEG monitoring, resulting in potential selection bias. The aim of this study is to develop hospital administrative data-based models to identify acute inpatient admissions with cEEG monitoring.

## Methods

### Study cohort

**Figure 1:**
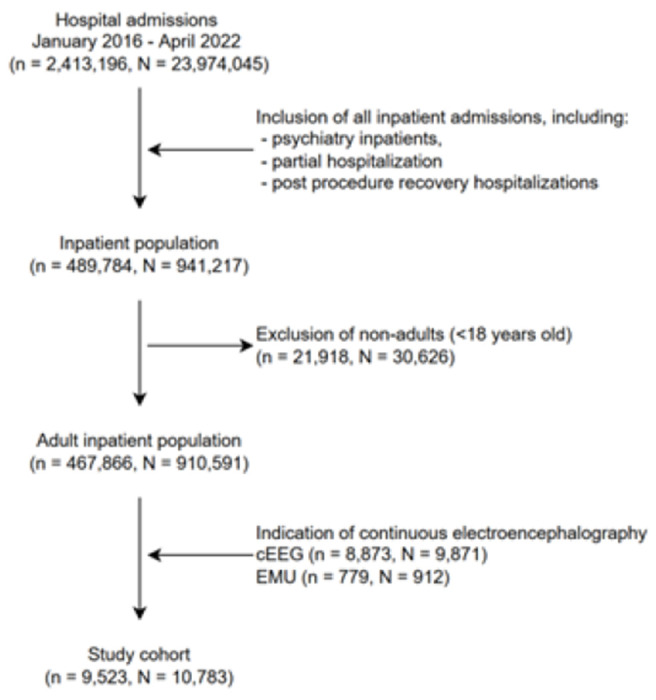
Study cohort inclusion and exclusion criteria. N – number of visits; n – number of patients; cEEG – acute inpatient hospitalizations admissions class; EMU – epilepsy monitoring unit admissions class.

In this study, we conducted a retrospective analysis of adult patients (≥ 18 years old) admitted to a single center between January 1st 2016 and April 30th 2022. The research protocol was approved by the Mass General Brigham (MGB) Institutional Review Board and a waiver of informed consent was obtained. The selection of patients for our cohort was performed considering the aim of the study in identifying acute inpatient admissions with cEEG monitoring. Figure 1 shows the patient selection flow chart. Patients were included if they underwent cEEG monitoring (either in the EMU or as part of an acute inpatient hospitalization). cEEG monitoring was defined using long-term EEG monitoring ICD10th revision and CPT codes, (Table A1 from the Additional File).

### Study outcome variables

Our study outcome consisted of a binary variable indicating whether an inpatient admission with a cEEG procedure was performed in the acute inpatient hospital setting (cEEG) or in the EMU setting (EMU). From here on “cEEG” will refer to acute inpatient admissions with continuous EEG monitoring, and “EMU” will refer to epilepsy monitoring unit admissions. The gold standard for the procedures was defined using the hospital EEG recording platform.

### Study covariates

The study covariates for the hospital admissions in our study cohort are presented in Table A2 from the Additional File. Diagnoses and procedures were defined using ICD and CPT codes and are presented in Table A1 from the Additional File. The binary covariates considered were indication (‘1’ for presence and ‘0’ for absence) of daily laboratory values acquired, inpatient medications ordered, procedures performed, type of admission – elective, emergency and urgent, primary and secondary diagnoses of traumatic brain injury (TBI), stroke and epilepsy, seizures or convulsions, death at discharge, discharged to home or self-care and female sex. The numerical covariates consisted of the number of distinct procedures, number of distinct medications, days of hospital length of stay (LOS) and age at admission. Numerical covariates were normalized using the min-max normalization (Han, Pei, and Kamber 2011) where the minimum and maximum reference values for each covariate were calculated from a training set. The data splitting into training and testing sets is detailed in the following section. Regarding outliers preprocessing, we identified one outlier for hospital LOS, which we imputed with the median LOS.

The procedures (Table A1 from the Additional File) considered were the following: abdomen/pelvis computerized tomography (CT) scan, arterial line, chest X-ray, head CT scan, lumbar puncture, magnetic resonance imaging (MRI), mechanical ventilation, transthoracic echocardiogram, and tube feed orders. The number of procedures consisted of the sum of the distinct procedures performed during the hospital stay, varying in the range between zero and nine.

The set of inpatient medications considered were the following: cefepime, ceftriaxone, dexmedetomidine, dobutamine, dopamine, enoxaparin, epinephrine, heparin, midazolam, nicardipine, norepinephrine, phenylephrine, piperacillin, piperacillin/tazobactam, propofol, vancomycin and vasopressin. The number of medications consisted of the sum of the distinct inpatient medications ordered during the hospital stay, varying in the range between zero and seventeen.

### Modeling design and evaluation

We performed a random sampling of hospital admissions in our cohort to create training (70%) and hold-out testing (30%) sets with distinct patients. With the training set we developed an extreme gradient boosting model (XGBoost) (Chen and Guestrin 2016) and performed hyperparameter tuning in 100 iterations of 10-fold cross validation. The hyperparameter tunning methodology is described in Additional File section A.2. We selected a threshold for binary classification on the training data that achieved a positive predictive value (PPV) yielding a balance between false positives and false negative predictions. We assessed both the positive and negative predictive values (PPV and NPV, respectively). We evaluated model performance using the area under the precision recall-curve (AUPRC) (Saito and Rehmsmeier 2015), showing the trade-off between PPV and sensitivity, also called true positive rate or recall, for different thresholds. We also evaluated the receiver operating characteristic (AUROC), which quantifies the tradeoff between sensitivity and false positive rate (also known as 1-specificity), across different decision thresholds (Steyerberg et al. 2010). Given the imbalance in our dataset, we present the macro average (Sokolova and Lapalme 2009) performance for the classification, and the performance for each class (EMU vs cEEG). A macro-average calculates performance metrics independently for both classes and then takes the average, giving both classes equal weight (Sokolova and Lapalme 2009). We performed 1000 bootstrapping iterations to calculate 95% confidence intervals (CI) in the hold-out test set, an external and independent test set not used for model training or validation. We assessed covariate importance using SHAP (SHapley Additive explanations) (Lundberg and Lee 2017), which estimates the contribution of each feature to the model’s predictions.

## Results

### Cohort characteristics

Our cohort comprised 9,523 patients and a total of 10,783 hospital admissions, after applying inclusion and exclusion criteria (Fig. 1). The average age of the cohort was 59 years (standard deviation (SD) 18.2), with the majority being males (53.8%), White (75.5%) and non-Hispanic (82.7%) (Table 1). The majority of admissions (91.5%) were acute inpatient hospitalizations (i.e., cEEG rather than EMU). Demographic characteristics were approximately the same at the hospital admission level (Table A2 from the Additional File; counting all admissions separately) as those at the patient level (Table 1; counting each patient only once).

### Modeling results

**Figure 2:**
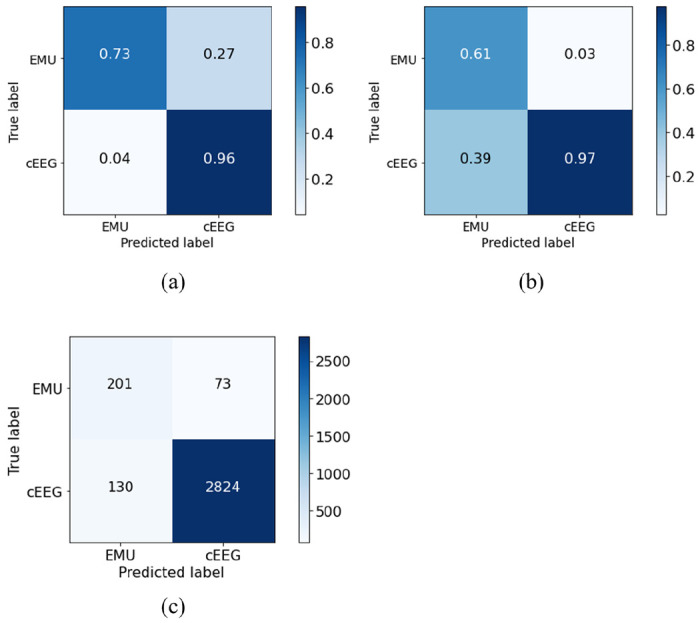
Confusion matrices normalized by (a) sensitivity, (b) positive predictive value, (c) without normalization. cEEG – acute inpatient hospitalizations admissions class; EMU – epilepsy monitoring unit admissions class.

The XGboost model was trained with all study covariates described in the Methods section (Table A2 from the Additional File). Model performance evaluated on the testing set is presented in Table 2. The hyperparameters selected during training in 10-fold cross validation are presented in Table A3 from the Additional File. We experimented different thresholds for fixed values of PPV between 95% and 98% (Table A4 from the Additional File). When setting PPV to 98%, the binary threshold was 0.80 and yielded a balanced sensitivity and specificity. The model achieved a macro AUROC of 0.92 (95% CI [0.91–0.94]) and AUPRC of 0.99 [0.98–0.99]. The AUROC and AUPRC curves are presented in Figure A1 from the Additional File. There were 130 (4%) misclassifications of acute inpatient cEEG incorrectly classified as EMU admissions, and 73 (26.6%) misclassifications of EMU admissions incorrectly classified as acute inpatient cEEG, as presented in Fig. 2. When analyzing misclassifications, 130 cEEG admissions incorrectly classified as EMU, we observed that 79% (N = 102) of admissions were elective and 90% (N = 117) discharged to home or self-care. For the EMU admissions incorrectly classified as acute inpatient cEEG we observed that 71% (N = 52) were emergency and 93% of the admissions (N = 68) had daily laboratory values acquired, a higher proportion when compared with that of the EMU class (59%, Table A2 from the Additional File).

Since most EMU admissions in our cohort are elective (72.4%) and discharged to home or self-care (82.7%), (Table A2 from the Additional File), we trained a model excluding both the admission type covariates (emergency, urgent and elective) and discharge disposition to home or self-care and evaluated the performance in test (AUROC and AUPRC presented in Figure A2 from the Additional File). The overall macro average model performance (Table A5 from the Additional File) was similar to that of the model using all covariates (Table 2) with an AUROC of 0.90 (95% CI [0.88–0.92]) and AUPRC of 0.99 [0.98–0.99]. However, this model showed a higher number of misclassifications, especially false positives, mainly due to errors in classifying the EMU class.

### Covariates importance

**Figure 3:**
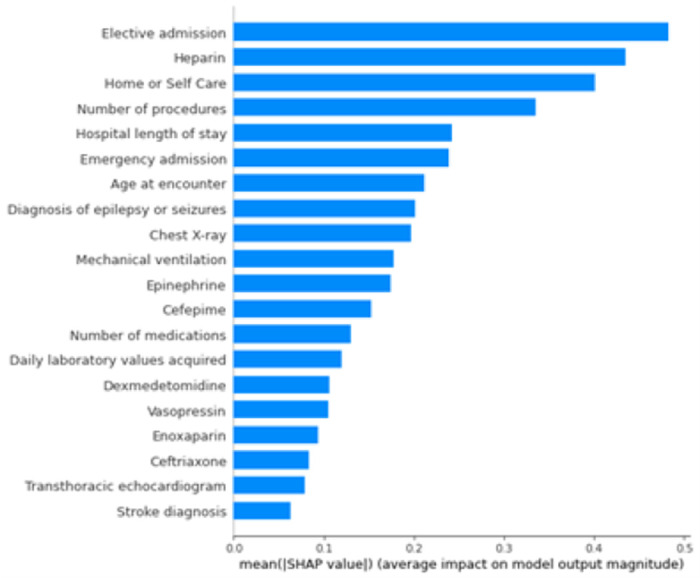
Average magnitude of the Shapley Additive exPlanations (SHAP) values for the model top 20 features. Positive SHAP values further from zero represent higher impact for the acute inpatient admissions class (cEEG) while negative SHAP represent higher impact for the epilepsy monitoring unit admissions (EMU) class.

We analyzed the importance of the covariates in the design of the XGBoost model. The average magnitude of the SHAP values for the 20 top features is presented in Fig. 3, and the SHAP raw values are presented in Figure A3 from the Additional File.

Elective admission was the most important covariate for the model to classify an admission as EMU (left side in Figure A3 from the Additional File). We sought to understand the type of admissions distribution for each class, since EMU admissions are frequently elective. For both train and test sets, 72% of EMU and 11% of acute inpatients (cEEG class) admissions were elective, respectively. Furthermore, we assessed if the EMU non-elective admissions were correlated with the COVID-19 pandemic. According to a study (Zepeda et al. 2021), in the setting of the COVID-19 pandemic, urgent and emergent EMU admissions were required due to increased seizure or event frequency. We confirmed that the non-elective EMU admissions spanned all years of our study period with the following number of admissions per year: 39, 46, 48, 41, 31, 38, 9, from 2016 to 2022, respectively. Since not all EMU admissions were elective, it was important to combine this covariate with others to develop the classification model.

Emergency admissions, orders of medications such as heparin, vasopressin, cefepime or epinephrine, daily laboratory values acquired, mechanical ventilation, transthoracic echocardiogram and chest X-ray and also diagnosis of stroke were important predictors of acute inpatients hospitalizations (cEEG).

The EMU class was associated with younger age at admission (blue color in Figure A3 from the Additional File) when compared to the cEEG class (mostly pink in Figure A3 from the Additional File). The average age (SD) for EMU admissions was 42 (18) while for the cEEG class it was 60 (18), a difference of approximately 20 years (Table A2 from the Additional File). The EMU admissions class was also associated with lower number of procedures and medications, when compared to the cEEG class. EMU admissions were also associated with being discharged home, having a diagnosis of epilepsy or seizures, orders of enoxaparin and ceftriaxone. Patients receiving ceftriaxone along were likely intracranial monitoring admissions.

## Discussion

Our model using hospital administrative and billing data distinguishes continuous EEG performed in acute inpatient setting from the EMU setting and can enable comprehensive comparative effectiveness and cost effectiveness analysis of continuous EEG utilization in the acute and critical care setting. Such large epidemiologic studies can then provide further guidance for randomized trials of continuous EEG guided anti-seizure treatment in the acute setting, and refinement of continuous EEG guidelines and protocols, particularly for resource limited settings.

There has been limited prior work in the development and validation of administrative models for accurate identification of continuous EEG in the hospital setting from administrative datasets. One prior study evaluated ICD based models for accurate identification of EMU admissions from administrative datasets (Kamitaki et al. 2020). The authors examined three queries, with varying use of admission and primary diagnosis ICD codes in 351 admissions. They found that queries combining ICD and CPT codes for continuous EEG, with ICD codes for epilepsy, seizure, or seizure mimic codes as the admitting diagnosis had a sensitivity of 96.3%, specificity of 100.0%, positive predictive value of 98.3%, and negative predictive value of 100.0%. Models combining ICD/CPT codes for continuous EEG and ICD codes for epilepsy and seizures as principal diagnosis had sensitivity of 94.9%, specificity of 100.0%, and positive and negative predictive values of 98.8%, and 100.0% respectively. However, these queries only included elective admissions, with focus only on EMU admissions and therefore cannot be applied for identification of acute inpatient continuous EEG utilization. Additionally, our work demonstrates that a subset of EMU admissions are emergent or urgent (up to 28% in our cohort), and a subset of acute inpatient cEEG admissions are elective (up to 11% in our cohort). Our model reduces the misclassification rate (4% cEEGs misclassified as EMU vs. 11% using a priori stratification on the elective vs. non elective admission status). We did not see a significant change in the misclassification of EMU (27% of EMU admissions misclassified as acute inpatient cEEG vs. 28% using a priori stratification on the elective vs. non elective admission status). While elective vs. emergent and urgent admissions, continues to be the most important predictors in our model, combining them with additional ICD diagnosis, procedure and medication codes can enable identification of acute inpatient cEEGs without a priori exclusion of patients.

Two prior studies have examined the impact of cEEG utilization in critically ill patients using the Nationwide Inpatient Sample (Hill et al. 2019; Ney et al. 2013). Both studies found that cEEG utilization is associated with lower in-hospital mortality, and is not associated with increased costs when compared with routine (brief) EEG (Hill et al. 2019; Ney et al. 2013). However, to ensure exclusion of EMU admission, the studies excluded all elective admissions. Additionally, to ensure the cohort was restricted to critically ill patients they only included patients that received mechanical ventilation. Our model can eliminate the need for upfront inclusion and exclusion criteria based on admission status, enabling identification of a broader more complete cohort of admissions with inpatient continuous EEG utilization from administrative datasets.

The main limitation of the study is that it is a single center study, therefore may not be generalizable. However, we used standard billing and procedure codes, medications and admission data that are routinely available in most large commercially available inpatient datasets and in hospital administrative datasets. Other covariates could have been included in the model, such as free text clinical notes, including EEG reports, which we propose as future work, along with validation in other administrative datasets.

## Conclusions

The model developed in this study can identify continuous EEG performed in the acute inpatient setting from continuous EEG performed in the EMU setting and reduces the number of misclassifications. This model will allow the identification of continuous EEG monitoring admission in larger cohorts, thereby contributing to the scale of research of EEG utilization.

## Figures and Tables

**Table 1:** Main characteristics of the study cohort.

Characteristic	Study cohort (n = 9523, N = 10783)
**Age**^(a)^, **(years, mean (SD))**	59 (18.2)
**Female sex, n (%)**	4402 (46.2)
**Race, n (%)**	
White	7182 (75.5)
Black or African American	818 (8.6)
Asian	329 (3.6)
Other^(b)^	1173 (12.3)
**Ethnicity, n (%)**	
Non-Hispanic	7879 (82.7)
Hispanic	701 (7.4)
Unknown	943 (9.9)
**Hospital admissions, N (%)**	
EMU	912 (8.5)
cEEG	9871 (91.5)
**Type of admission, N (%)**	
Emergency	6902 (64.0)
Urgent	2146 (20.0)
Elective	1729 (16.0)
**Discharge disposition, N (%)**	
Deceased	1897 (17.6)
Home or Self Care	2812 (26.1)
**Diagnosis, N (%)**	
TBI	698 (6.5)
Stroke	1507 (14.0)
Epilepsy, seizures or convulsions	6876 (63.8)
**Daily laboratory values acquired, N (%)**	10127 (93.9)
**Top procedures, N (%)**	
Chest X-ray	7052 (65.4)
Head CT scan	6546 (60.7)
MRI	5869 (54.4)
**Top medications, N (%)**	
Enoxaparin	7137 (66.2)
Propofol	6067 (56.3)
Vancomycin	5777 (53.6)
**Medications**^(b)^, **(number, median [IQR])**	5 [1, 8]
**Procedures**^(b)^), **(number, median [IQR])**	3 [1, 5]
**LOS (days, median [IQR])**	9 [5, 19]

cEEG – acute inpatient hospitalizations admissions class; EMU – epilepsy monitoring unit admissions class; LOS – hospital length of stay; N – number of hospital admissions; n – number of patients.

(a) Age at baseline for the first hospital admission in the study period.

(b) ‘Other’ includes ‘unknown’, ‘declined’, ‘American Indian or Alaska Native’ and ‘Native Hawaiian or other Pacific Islander’.

**Table 2:** Modeling performance [95% confidence intervals] of the extreme gradient boosting model evaluated test.

Classes	AUROC	AUPRC	Sensitivity	PPV	NPV	Specificity
Macro average	0.92 [0.91–0.94]	0.98 [0.98–0.99]	0.78 [0.75–0.81]	0.83 [0.79–0.85]	0.78 [0.79–0.85]	0.78 [0.75–0.81]
EMU	0.92 [0.91–0.94]	0.66 [0.60–0.72]	0.73 [0.68–0.79]	0.61 [0.55–0.65]	0.97 [0.97–0.98]	0.96 [0.95–0.96]
cEEG	0.92 [0.91–0.94]	0.99 [0.98–0.99]	0.96 [0.95–0.96]	0.97 [0.97–0.98]	0.61 [0.55–0.65]	0.73 [0.68–0.79]

AUROC – area under the receiver operating characteristic curve; AUPRC – area under the precision-recall curve; cEEG – acute inpatient hospitalizations admissions class; EMU – epilepsy monitoring unit admissions class; PPV – positive predictive value; NPV – negative predictive value.

## Data Availability

We will made our de-identified data and code available in a public GitHub repository for reproducibility, upon article acceptance for publication.
